# Breast tuberculosis: a report of five cases

**DOI:** 10.1186/s41182-017-0081-6

**Published:** 2017-12-14

**Authors:** Boubacar Efared, Ibrahim S. Sidibé, Fatimazahra Erregad, Nawal Hammas, Laila Chbani, Hinde El Fatemi

**Affiliations:** 1 Department of Pathology, Hassan II University Hospital, Fès, Morocco; 2 Faculty of Medicine and Pharmacology, Sidi Mohamed Ben Abdellah University, Fès, Morocco

**Keywords:** Breast, Tuberculosis, Caseous necrosis, Endemic, Morocco

## Abstract

**Background:**

Breast tuberculosis is a rare form of extrapulmonary tuberculosis with clinical and radiological misleading presentations. We report herein a retrospective study of clinicopathological features of five cases of breast tuberculosis collected at Hassan II University Hospital of Fès, Morocco, a country where tuberculosis is endemic.

**Case presentation:**

The mean age was 40.6 years (range of 21–59 years). Two patients presented with abscessed lesions, and three cases presented with breast lumps with a suspicion of malignancy on imaging techniques. The diagnosis has been made on histological specimens (3 biopsy specimens, 1 excisional biopsy, and 1 lumpectomy). All patients have been successfully treated after the completion of the standard antibiotherapy.

**Conclusions:**

Our current study shows that the breast is rarely affected by tuberculosis even in endemic area. The clinical presentation is often misleading, and the histopathological analysis constitutes a valuable diagnostic tool. The prognosis of breast tuberculosis is good after treatment by a standard antibiotherapy.

## Background

Breast tuberculosis (BTB) is a very rare form of extrapulmonary tuberculosis (EPTB) even in endemic areas of the world [1, 2]. The incidence of BTB ranges from 0.1% in developed countries to 4.5% in endemic countries [2, 3]. The breast gland, as well as the spleen or the skeletal muscle, is a hostile milieu for the development of the *Mycobacterium (M.) tuberculosis*, the main causative agent of tuberculosis (TB) [2]. Breast tuberculosis affects commonly women in reproductive age and the clinical presentation is often misleading, mimicking a pyogenic abscess or a malignant breast tumor [3, 4], hence the need of a correct diagnosis for an appropriate management of patients. The radiological techniques are not always accurate, as BTB can also mimic a malignant tumor [3, 5, 6]. Biological techniques, such as pus culture or the search for acid-fast bacilli by Ziehl-Neelsen staining, are less sensitive and time-consuming [1, 2]. The polymerase chain reaction (PCR) technique is very sensitive, but expensive and not affordable in poor areas where TB is endemic [2].

The histopathological examination provides usually the accurate diagnosis of TB by showing typical necrotizing granulomas and by excluding all differential diagnosis especially malignant tumors [1, 2, 7]. The management of BTB relies mainly on a standard antibiotherapy combining many drugs, and the surgical treatment is rarely required [2, 7].

The aim of our current study is to report about a rare location of a common disease (breast tuberculosis) and to highlight the major diagnostic role of the pathology in this rare location, in order to provide appropriate management for the patients.

## Case presentation

**Fig. 1:**
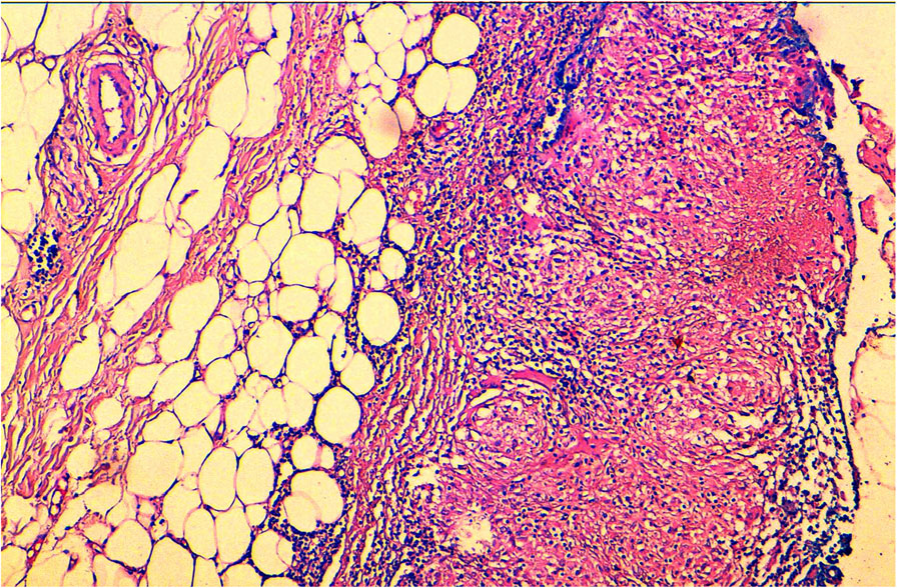
Histological image of breast tuberculosis showing a necrotizing granulomatous lesion (hematoxylin eosin stain × 100) (patient 5)

**Fig. 2:**
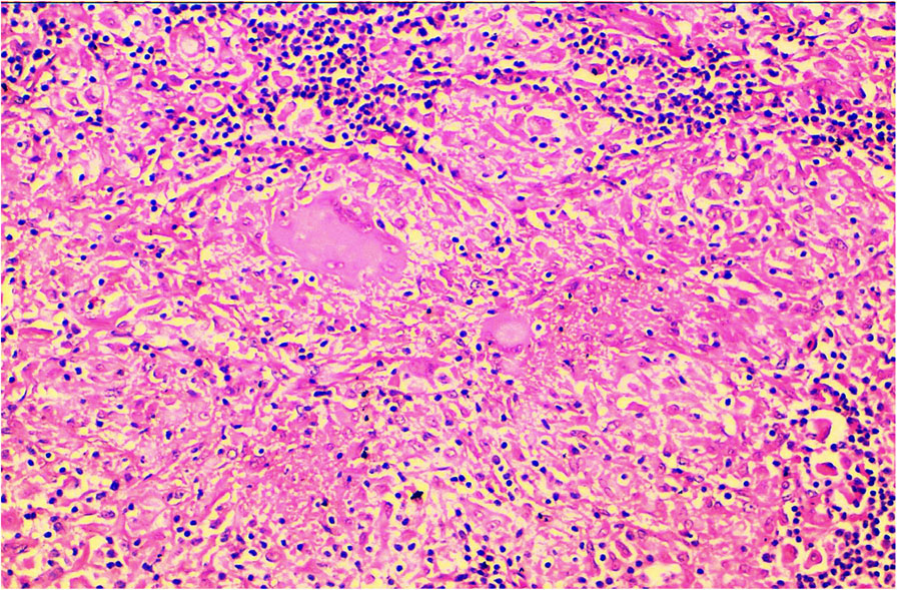
A higher magnification showing areas of caseous necrosis, epithelioid cells, and giant multinucleated cells, surrounded by lymphocytes (hematoxylin eosin stain × 200)

Medical records of all patients histologically diagnosed with breast tuberculosis were retrospectively collected at the Department of Pathology of Hassan II University Hospital of Fès, Morocco (from 2006 to 2017). The diagnosis of BTB was rendered when the histological examination showed typical lesions consisting of granulomas associated with typical caseous necrosis (Figs. 1 and 2). Cases with granulomatous lesions without caseous necrosis have been excluded.

The mean age of our five cases was 40.6 years (range of 21–59 years), and all patients were females. All cases had unilateral BTB, with the left side mostly affected (3 cases out of 5) (Table 1). Two patients presented with abscessed lesions, while the remaining three cases presented with breast lumps and a suspicion of malignancy on imaging techniques with lesions categorized as BI-RADS 4 (breast imaging reporting and data system). Of these patients, two had concomitant axillary lymphadenopathy (patients 4 and 5). The histological diagnosis has been made on three biopsy specimens, one excisional biopsy specimen, and one lumpectomy specimen. The diagnosis of TB on axillary lymph nodes (LN) has been performed on biopsy for patient 4 and on fine-needle aspiration (FNA) for patient 5.

**Table 1:** Clinicopathological features of our patients diagnosed with breast tuberculosis

Cases	Age/gender (years)	Clinical presentation	Other involved sites	Suspicion of malignancy	Side	Specimens	Treatment
1	40/F	Abscess	–	–	Left	Biopsy	Drainage + ATB
2	38/F	Lump	–	BI-RADS 4	Left	Biopsy	ATB
3	21/F	Abscess-fistula	–	–	Right	Biopsy	Drainage + ATB
4	45/F	Lump	Axillary LN	BI-RADS 4	Left	Biopsy	ATB
5	59/F	Lump	Axillary LN	BI-RADS 4	Right	Lumpectomy	Surgery + ATB

*F* female, *LN* lymph node, *BI-RADS* breast imaging reporting and data system, *ATB* antibiotherapy

All patients have received the standard antibiotherapy consisting of a combination of antibiotics (2 months of rifampicin, isoniazid, and pyrazinamide, followed by 4 months of rifampicin and isoniazid). Surgical drainage has been performed for patients with breast abscess (patients 1 and 2). Surgical treatment has been performed (lumpectomy) for patient 5. All patients have been successfully treated after the completion of the 6 months standard ATB, with no residual disease.

## Discussion

We report herein cases of BTB in Fès, Morocco, an endemic country where the incidence of TB in 2008 was 81 per 100,000 overall, with 28,000 new cases each year [8, 9]. Breast tuberculosis is very rare even in endemic countries [2, 5]. The breast gland is not an ideal site for the survival and multiplication of *Mycobacterium tuberculosis* [2]. This bacterium seems to reach the breast by several ways: lymphatic or hematogenous route and direct inoculation from adjacent structures (ribs, pleura, lung) [1–4]. The inoculation by retrograde lymphatic route seems to be a more suitable theory as often patients with BTB have an associated lymph node TB [3, 6]. In our series, two patients (40%) had concomitant axillary lymph node TB. In a large series of 46 cases of BTB, Kilic et al. have found eight cases (17.4%) with axillary lymphadenopathy [3].

The clinical presentation of BTB is usually associated with breast pain, breast nodule, abscess, or nipple discharge [1, 3, 6]. More often, BTB affects women in reproductive age, men are very rarely affected [2, 7]. The disease is often unilateral, very rarely bilateral [3]. Patients with breast nodules often present with misleading clinical and radiological aspects, with suspicion of malignancy [3, 4, 6]. Kilic et al. reported in their series that 34.8 and 43.5% of patients presented respectively with clinical and radiological suspicion of malignancy (BI-RADS 4 or 5) [3]. In our cases, three patients (60%) presented with radiological suspicion of malignancy (BI-RADS 4).

For adequate management, a correct diagnosis of BTB should be performed. Biological methods, such as acid-fast stain (Ziehl-Neelsen stain) or *M. tuberculosis* culture, lack sensitivity because BTB lesions are often pauci-bacillary [1, 2, 10]. Molecular techniques like polymerase chain reaction (PCR) are very sensitive but are expensive and not affordable in poor areas where TB is endemic [2]. Pathological examination seems to be more sensitive and less expensive and less time-consuming. The core needle biopsy is more sensitive than cytological method (fine needle aspiration) [2, 10]. However, the histopathological examination of BTB should show typical TB lesions consisting of granulomas associated with typical caseous necrosis. Differential histological diagnoses include several breast lesions that can harbor granulomatous lesions without caseous necrosis: idiopathic granulomatous mastitis, breast sarcoidosis, Wegener granulomatosis, etc. [2]. In our series, all five cases showed granulomas with typical caseous necrosis. The biopsy in one patient (patient 5) failed at first to show caseous necrosis, but it has been repeated and then showed typical TB lesions.

The management of BTB relies mainly on a standard antibiotherapy combining many drugs (classically, 2 months of rifampicin, isoniazid, and pyrazinamide with or without ethambutol, followed by 4 months of rifampicin and isoniazid). A surgical treatment is associated when there are abscesses or important tissue destruction [1–3, 7, 11].

## Conclusion

Breast tuberculosis is a very rare form of extrapulmonary tuberculosis even in endemic areas. The diagnosis is challenging as clinical and radiological presentations are misleading. Pathology is a valuable diagnostic tool that can achieve a correct diagnosis as sometimes biological techniques (culture, PCR) are time-consuming, more expensive, and not conclusive.

## Abbreviations

ATB: Antibiotherapy
BI-RADS: Breast imaging reporting and data system
BTB: Breast tuberculosis
TB: Tuberculosis
